# “Expectant Parents”: Study protocol of a longitudinal study concerning prenatal (risk) factors and postnatal infant development, parenting, and parent-infant relationships

**DOI:** 10.1186/1471-2393-12-46

**Published:** 2012-06-11

**Authors:** A Janneke BM Maas, Charlotte MJM Vreeswijk, Evi SA de Cock, Catharina HAM Rijk, Hedwig JA van Bakel

**Affiliations:** 1 Department of Developmental Psychology, Tilburg University, Room P-704, P.O. Box 90153, 5000, Tilburg, the Netherlands; 2 Centre of Infant Mental Health, Dimence, Deventer, the Netherlands

**Keywords:** Attachment, Bonding, Mother, Father, Pregnancy, Risk factors, Representations, Infant

## Abstract

**Background:**

While the importance of the infant-parent relationship from the child’s perspective is acknowledged worldwide, there is still a lack of knowledge about predictors and long-term benefits or consequences of the quality of parent-infant relationships from the parent’s perspective. The purpose of this prospective study is to investigate the quality of parent-infant relationships from parents’ perspectives, both in the prenatal and postpartum period. This study therefore focuses on prenatal (risk) factors that may influence the quality of pre- and postnatal bonding, the transition to parenthood, and bonding as a process within families with young children. In contrast to most research concerning pregnancy and infant development, not only the roles and experiences of mothers during pregnancy and the first two years of infants’ lives are studied, but also those of fathers.

**Methods/design:**

The present study is a prospective longitudinal cohort study, in which pregnant women (N = 466) and their partners (N = 319) are followed from 15 weeks gestation until their child is 24 months old. During pregnancy, midwives register the presence of prenatal risk factors and provide obstetric information after the child’s birth. Parental characteristics are investigated using self-report questionnaires at 15, 26, and 36 weeks gestational age and at 4, 6, 12, and 24 months postpartum. At 26 weeks of pregnancy and at 6 months postpartum, parents are interviewed concerning their representations of the (unborn) child. At 6 months postpartum, the mother-child interaction is observed in several situations within the home setting. When children are 4, 6, 12, and 24 months old, parents also completed questionnaires concerning the child’s (social-emotional) development and the parent-child relationship. Additionally, at 12 months information about the child’s physical development and well-being during the first year of life is retrieved from National Health Care Centres.

**Discussion:**

The results of this study may contribute to early identification of families at risk for adverse parent-infant relationships, infant development, or parenting. Thereby this study will be relevant for the development of policy, practice, and theory concerning infant mental health.

## Background

Developmental research has firmly established the quality of the relationship between an infant and his or her parent as an important factor influencing the child’s later development [1-6]. When children develop a secure relationship with their parents or caregivers in their first years of life, they generally have better cognitive outcomes, better social interactions, display less behavioral problems, and achieve better at school [7]. Research in this area has mainly investigated the attachment relationships that infants form with their parents, thus focusing on the child’s perspective of the relationship. In contrast, the attachment relationship from the parent’s perspective has not been frequently studied. This concept, also known as *bonding*, may be of equal importance to later child development as the traditionally studied concept of infant-to-parent attachment. More research concerning predictors and long-term benefits or consequences of bonding is therefore needed [8].

The development of the parent-infant attachment relationship does not start after the child is born, but already evolves during pregnancy [9,10]. The relationship a parent forms with the fetus is often referred to as *prenatal attachment* and has been described as the earliest, most basic form of human intimacy [11]. Several definitions of prenatal attachment have been provided, many conceptualized in health research, but it is generally defined as the emotional tie or bond that develops between expectant parents and their fetus [12,13]. Researchers have pointed out that it is important to study prenatal attachment and factors related to its development, since it provides insightful information on later parent-infant bonding [11]. Several studies found that the quality of the parent-fetus relationship was related to the quality of postnatal parent-infant relationships [14-17]. It is assumed that the prenatal parent-infant relationship influences the parent’s daily interactions with the child after birth and subsequently affects the quality of the parent-infant relationship and development.

Next to these feelings of attachment during pregnancy, research concerning the parent-fetus relationship has focused on another concept known as *internal working models* or *representations* of the unborn child [18,19]. Representations are described as a set of tendencies to behave in particular ways in intimate relationships [20]. They provide information about the ‘meaning’ a child has to his or her parent by asking the parent about his or her experiences with and perceptions of the fetus, (future) parenting, and the relationship with the fetus. The majority of research concerning internal working models has focused on postnatal representations, while studies on parents’ prenatal representations are scarce. Since prenatal representations are found to be related to postnatal representations and postnatal parent-infant interaction, it is important that the quality of prenatal representations and its consequences are also further investigated [14,17,21]. In addition, it is unknown whether discrepancies between pre- and postnatal representations lead to parental adjustment problems once the child is born, possibly affecting the quality of postnatal bonding and later child outcomes.

Parent-infant attachment or bonding develops further after birth and continues to develop beyond the early postnatal period [22]. Surprisingly, empirical research into the determinants, consequences, and stability of postnatal bonding is also limited [23]. Only a few studies have examined predictors and consequences of postnatal bonding and they suggest that prematurity, domestic violence during pregnancy, and maternal postpartum mood are related to adverse maternal bonding and adverse parent-infant interactions [24-27]. Moreover, Brockington [28] stressed that both severe disturbances, as well as less severe problems with parental bonding may lead to more negative parental care and may subsequently result in various forms of child abuse or neglect. Therefore, several parental, infant, and contextual risk factors are expected to influence the quality of the bonding process.

The present study has been designed to investigate prenatal (risk) factors that may influence the quality of pre- and postnatal parent-infant relationships and postnatal infant development within families with young children. Several determinants and consequences of the early parent-infant relationship will be investigated. Already during pregnancy, prenatal risk factors influencing the quality of the parent-infant relationship and later child development can be identified [29]. For example, emotional problems of mothers during pregnancy, problems in mothers’ own childhood history, and deficits in parental cognitive functioning increase the possibility of problematic caregiving and child development [30,31]. However, there is still considerable debate and a lack of knowledge about how specific risk factors are related to the long-term benefits or consequences of the parent-infant relationship. In contrast to most research concerning pregnancy and infant development, this study does not only focus on maternal characteristics, but also on the roles and experiences of fathers during pregnancy and the first two years of the infants’ lives.

The following topics and research questions related to the parent-infant relationship will therefore be investigated in the current study:

1)The relationship between prenatal (risk) factors, postnatal infant development and quality of the parent-infant relationship. Can specific prenatal risk factors for adverse infant development, parenting, or parent-infant relationships be identified during pregnancy?

2)The transition to parenthood. Is there a discrepancy between the quality of prenatal and postnatal parent-infant relationships and parents’ representations of the child? Do parents’ prenatal expectations of the child’s characteristics meet their postnatal experiences? How are these factors related to infant behavior and development?

3)Parental bonding over time. What are the stability and change in parents’ feelings of bonding over time? Is the quality of early parent-infant bonding related to later child development?

## Methods/design

### Enrollment and informed consent

Between November 2008 and July 2009, 835 pregnant women were invited by their midwives to participate in this study. Four midwifery practices in Eindhoven, the 5^th^ largest city of the Netherlands, agreed to participate in the study. At the first routine visit (between 9–15 weeks gestational age), midwives gave mothers information about the purpose of the study and invited them to participate. The oral information was accompanied by an information brochure with specific information about the study, which each mother received. If mothers were interested in participation, one of the researchers contacted them by phone to provide additional information and asked whether mothers wanted to enroll in the study. Partners were not directly approached by the researchers but the mothers were informed about the importance of involvement of their partners in the study. After parents received oral and written information about the protocol, both parents were asked for written consent. The informed consent form consisted of three different options. Parents could consent to (1) active participation in the complete research protocol, including two home visits, (2) active participation by filling in questionnaires but not by participating in home visits, or (3) passive participation by allowing the researchers to gather information from the midwife and National Health Care Centres, but without home visits or filling in questionnaires. Separate informed consent forms were sent to mothers and their partners. Once parents returned the signed forms, enrollment in the study was complete.

The “Expectant Parents” [“In Verwachting”] study protocol has been financed and approved by the Netherlands Organization for Health Research and Development (ZonMW, Grant 80-82405-98-074/157001020). It was also approved by the Medical Ethics Committee of St. Elisabeth Hospital Tilburg (date: 13-08-2008, register number: NL 23376.008.08).

### Participants

**Figure 1:**
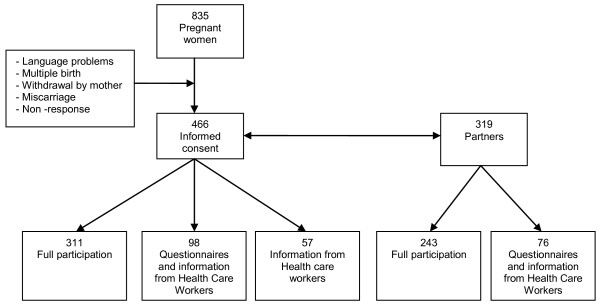
Flow chart of study population ‘Expectant Parents’.

Of the 835 invited women, women with a poor understanding of the Dutch and English language, those expecting multiple births, and women who were over 20 weeks of gestation at enrollment, were excluded from participation. Reasons for not giving written consent were withdrawal by the mother, miscarriage, and non-responding mothers. As Figure 1 demonstrates, this resulted in 466 completed informed consent forms of expectant mothers and 319 informed consents of their partners. All parents hereby gave permission to the researchers to retrieve information about the pregnancy and delivery from their midwives and information about the development of the child in the first year of life from National Health Care Centres. Of these parents, 409 mothers and 319 fathers agreed to complete questionnaires, of which 311 mothers and 243 fathers also agreed to participate during home visits (full participation).

### Study design

**Figure 2:**
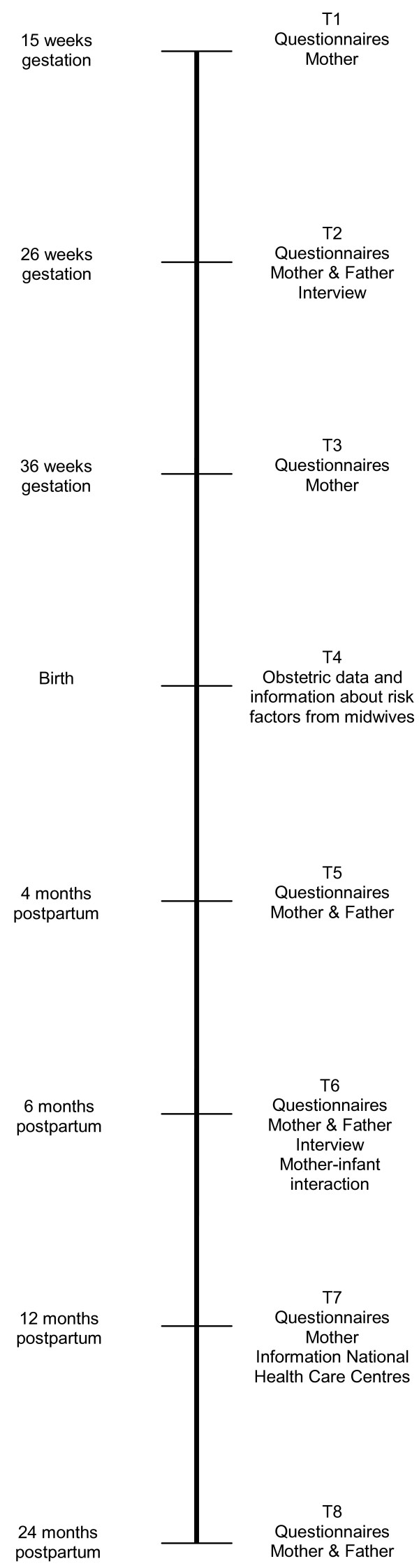
Time line study protocol ‘Expectant Parents’.

The present study is a prospective longitudinal cohort study, in which pregnant women and their partners were followed from 15 weeks gestation until their child was 24 months old. As can be seen in Figure 2, pregnant women completed questionnaires at 15, 26, and 36 weeks gestational age. At 26 weeks of pregnancy, their partners also completed a questionnaire. At the same time a home visit took place during which a standardized interview concerning the prenatal representations of the unborn child was conducted with both parents separately.

Postnatally, there were five more measurement waves (at birth and at 4, 6, 12, and 24 months postpartum). Obstetric information about the birth of the child, including birth weight, Apgar score, and possible complications was registered by the midwives in line with their general practice guidelines. Additionally, midwives provided information about the presence of possible prenatal risk factors within families by completing an adapted Dutch version of the Dunedin Family Services Indicator (DFSI) [31]. At 4 and 6 months postnatally, both parents received questionnaires. At the child’s age of 6 months, an interview about the representations of their child was administered with both parents at their home, and the mother-child interaction was observed in several contexts within the home. Interviews generally lasted between 30 and 60 minutes and the observation of mother-infant interactions lasted approximately 20 minutes. All home visits were video-recorded. When children were 12 months old, mothers completed questionnaires concerning the child’s (social-emotional) development and information about the child’s physical development and well-being during the first year of life was retrieved from National Health Care Centres. At the child’s age of 24 months, the last measures concerning parental characteristics, the parent–child relationship, and the child’s development were completed by both parents.

### Study measures

**Figure 3:**
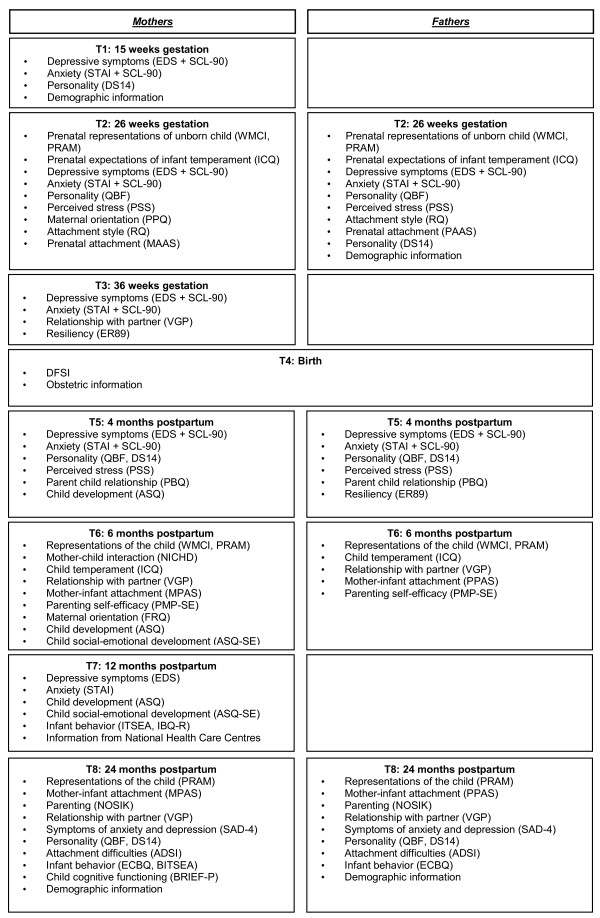
Study protocol and assessments at different time points during the study.

Figure 3 shows which variables were investigated at different time points during the study. Generally, the study measures can be classified according to whether they concern parental characteristics, infant characteristics, or the parent-infant relationship. Therefore, the selected instruments are described below according to these categories.

#### Parental characteristics

Parental characteristics were investigated using self-report questionnaires. To assess parental psychological well-being, the following questionnaires were used: Edinburgh Depression Scale (EDS) [32], State-Trait Anxiety Inventory (STAI) [33], Symptom Check List; anxiety, depression, and hostility subscale (SCL-90) [34], Symptoms of Anxiety-Depression index (SAD-4) [35], and Perceived Stress Scale (PSS) [36].

To assess parents’ personality characteristics, the Quick Big Five (QBF) [37], Type D Scale (DS14) [38], and Ego Resiliency 89 Scale (ER89) [39] were administered. Adult attachment style was measured with the Relationship Questionnaire Clinical Version (RQ-CV) [40] and the partner-relationship was evaluated with a subscale of the Questionnaire on Family Problems (Vragenlijst voor Gezinsproblemen; VGP) [41].

The Placenta Paradigm Questionnaire (PPQ) [10] and the Facilitator Regulator Questionnaire (FRQ) [42] were used to determine maternal orientations on pregnancy and their infants. Midwives used an adapted version of the Dunedin Family Services Indicator (DFSI) [31] to register the presence of possible prenatal risk factors among parents.

#### Infant characteristics

The following questionnaires were used to investigate infant development and behavior: Ages and Stages Questionnaire (ASQ) [43], Ages and Stages Questionnaire; Social-Emotional (ASQ-SE) [44], Infant Characteristics Questionnaire (ICQ) [45], Infant Toddler Social-Emotional Assessment (ITSEA) [46], Brief Infant Toddler Social-Emotional Assessment (BITSEA) [47], subscales of the Infant Behavior Questionnaire Revised (IBQ-R) [48], Early Childhood Behavior Questionnaire (ECBQ) [49], and Behavior Rating Inventory of Executive Function Preschool version (BRIEF-P) [50].

Information about the child’s physical development and well-being during the first year of life was retrieved from National Health Care Centres.

#### Parent-infant relationship

To determine parents’ representations of their (unborn) infant, the Working Model of the Child Interview (WMCI) [51] was conducted during home visits. At the same time and also at 24 months, the Pictorial Representations of Attachment Measure (PRAM) [52], a non-verbal measure of the parent-infant relationship, was administered. To evaluate the quality of mother-infant interactions, the NICHD scales [53] were used.

In addition, the following questionnaires were used to give insight into the parent-fetus and parent–child relationship: Maternal Antenatal Attachment Scale (MAAS) [12], Maternal Postnatal Attachment Scale (MPAS) [54], Paternal Antenatal Attachment Scale (PAAS) [12], Paternal Postnatal Attachment Scale (PPAS) [55], Parental Bonding Questionnaire (PBQ) [56], and Attachment Difficulties Screening Instrument (ADSI) [57].

To evaluate parenting behavior, the following scales were used: the Parental Stress Index (Nijmeegse Ouderlijke Stress Index-verkort; NOSI-K) [58], and Perceived Maternal Parenting Self Efficacy (PMP-SE) [59].

### Data collection and management

The logistics of this study were carried out by three researchers (AM, CV, EdC) in close collaboration with the midwives participating in this study. Before starting data collection, a protocol was set up and discussed with participating midwives to ensure that a uniform protocol was followed by all midwifery practices. Participating midwives were instructed on how to recruit pregnant women for participation in the study and how to register the presence of possible prenatal risk factors.

Questionnaires were sent to parents one or two weeks before the time point they should be completed or before the home-visits. All questionnaires were available in Dutch and English. Reminders were sent when parents failed to return the questionnaires. Table 1 shows the number of parents that participated at each measurement wave.

**Table 1:** Number of participants per time point of the study protocol

**Time**	**Measure**	**Mothers**	**Fathers**
T1: 15 weeks gestation	Questionnaires	406	-
T2: 26 weeks gestation	Questionnaires	375	299
	Home visit	311	243
T3: 36 weeks gestation	Questionnaires	351	-
T4: Birth	Information concerning the birth	455	-
	DFSI completed by midwife	445	-
T5: 4 months postpartum	Questionnaires	354	274
T6: 6 months postpartum	Questionnaires	341	268
	Home visit	295	225
T7: 12 months postpartum	Questionnaires	299	-
	National Health Care Centres	a	-
T8: 24 months postpartum	Questionnaires	248^b^	186^b^

Note.

a Data currently not complete.

b For T8, 285 mothers and 246 fathers were approached.

The researchers (AM, CV, CR) and several research assistants were trained to administer and code the WMCI, concerning parents’ representations of their (unborn) children and to code observations of mother-child interactions. All interviews and mother-infant interactions were video-recorded and coded afterwards. A random subgroup of the interviews and observations was coded by more than one coder to determine inter-rater reliability.

### Data preparation

Collected data were entered into an electronic database. Random samples of all manually processed questionnaires were double checked by the researchers to monitor the quality of the manual data entry. All measurements were checked by examination of the data, including their ranges, distributions, means, standard deviations, outliers, and logical errors.

### Privacy protection

Databases needed for answering specific research questions were centrally built from databases concerning different time points of the study. All information enabling identification of participants was erased from these databases, except identification numbers of each participant. Video-recordings of participants were stored on the computers of the researchers, which are only accessible with a password and not on web-based directories.

### Statistical analyses/power calculation

To answer the various research questions we will use structural equation modeling, regression analyses (HMR analyses), logistic regression analyses, and odds-ratio’s. Mediation and moderation analyses will follow Baron and Kenny’s requirements [60]. The power calculation is based on one of the main questions that will be addressed about the effects of prenatal (risk) factors on infant development. Assuming a moderate effect size of .30 or .40, a power of .80 (i.e., the minimal power for a similar study by Cohen [61]), an alpha of .05, and 11 parameters/predictors, we need a sample size of 220 participants (the power will be .83 with p = 11, *r² = .*09 or the power will be .99 with p = 11, *r²* = .16). Abovementioned power calculations are exact calculations, based on results of Gatsonis and Sampson [62]. Allowing for loss to follow-up by 24 months postpartum, we estimated that a sample of at least 240–260 women would be sufficient to test our hypotheses.

## Discussion

With this study we aim to gain more insight into the relationships between prenatal (risk) factors, postnatal infant development and the quality of the pre- and postnatal parent-infant relationship. This investigation will lead to more knowledge about the transition to parenthood for both mothers and fathers, and the stability and change in parents’ feelings of bonding over time. The longitudinal design with a multi-informant, multimethod approach offers the possibility to predict infant developmental outcomes in the first years of life from pregnancy onwards.

## Competing interests

The authors declare that they have no competing interests.

## Authors’ contributions

The study protocol was developed by HvB, in collaboration with AM, CV, EdC, and CR at the department of Developmental Psychology, Tilburg University, Tilburg, the Netherlands. AM and CV were appointed as PhD-students in 2008 and executed the study until children were 12 months old. These authors contributed equally to this work. EdC was appointed to the project as a PhD-student in a later phase and executed the study until children were 24 months old. All collaborators are considered as co-authors as they have significantly contributed to developing this research, obtaining the data, and writing the manuscript. All authors read and approved the final manuscript.

## Authors’ information

AM, CV, EdC, CR: Department of Developmental Psychology, Tilburg University, Tilburg, the Netherlands. HvB: Department of Developmental Psychology, Tilburg University, Tilburg, the Netherlands and Centre of Infant Mental Health, Dimence, Deventer, the Netherlands.

## Pre-publication history

The pre-publication history for this paper can be accessed here:

http://www.biomedcentral.com/1471-2393/12/46/prepub
